# The assessment of depression in people with multiple sclerosis: a systematic review of psychometric validation studies

**DOI:** 10.1186/s12888-016-0931-5

**Published:** 2016-08-04

**Authors:** Daniel Hind, Daphne Kaklamanou, Dan Beever, Rosie Webster, Ellen Lee, Michael Barkham, Cindy Cooper

**Affiliations:** 1 Clinical Trials Research Unit, School of Health and Related Research, University of Sheffield, Sheffield, UK; 2 Department of Psychology, Sociology and Politics, Sheffield Hallam University, Heart of the Campus, 42 Collegiate Crescent, Sheffield, S10 2BQ, UK; 3 Breast Cancer Now, London, UK; 4 Centre for Psychological Services Research, Department of Psychology, University of Sheffield, Sheffield, UK

**Keywords:** Depression, Multiple Sclerosis, Reproducibility of Results, Psychometrics, Chronic Disease

## Abstract

**Background:**

The prevalence of depression in people with multiple sclerosis (PwMS) is high; however, symptoms common to both conditions makes measurement difficult. There is no high quality overview of validation studies to guide the choice of depression inventory for this population.

**Methods:**

A systematic review of studies validating the use of generic depression inventories in people with MS was conducted using MEDLINE and PsycINFO. Studies validating the use of depression inventories in PwMS and published in English were included; validation studies of tests for cognitive function and general mental health were excluded. Eligible studies were then quality assessed using the COSMIN checklist and findings synthesised narratively by instrument and validity domain.

**Results:**

Twenty-one studies (*N =* 5,991 PwMS) evaluating 12 instruments were included in the review. Risk of bias varied greatly between instrument and validity domain.

**Conclusions:**

The review of validation studies was constrained by poor quality reporting and outcome reporting bias. Well-conducted evaluations of some instruments are unavailable for some validity domains. This systematic review provides an evidence base for trade-offs in the selection of an instrument for assessing self-reported symptoms of depression in research or clinical practice involving people with MS. We make detailed and specific recommendations for where further research is needed.

**Trial Registration:**

PROSPERO CRD42014010597

## Background

Multiple sclerosis (MS) is a chronic condition affecting the central nervous system. It is characterised by neurological symptoms and deficits that lead to increased disability and physical decline over 30–40 years [1]. Symptoms include fatigue, weakness, pain, cognitive impairment, loss of vision, tremors, poor balance, and bladder, bowel, and sexual dysfunction [2]. The prevalence of depression, which is strongly linked with a reduced quality of life, is high amongst people with MS (PwMS) with around half having major depression at some point in their lifetime and up to 40 % experiencing it at any one time [3, 4].

However, there is significant overlap in the somatic symptoms common to depression and MS, principally fatigue, pain, poor sleep and concentration, leading to concerns over the measurement of depression in PwMS [5]. For instance, the Diagnostic and Statistical Manual [6] criteria for depression includes common symptoms of MS such as fatigue, poor concentration and sleep difficulties. Other self-report measures of depressive symptoms include questions about health and work difficulties, which are also common to MS [7]. As such, levels of depression in MS may easily be overestimated, particularly when using self-report measures. The debate over how to assesses depression in the presence of a physical condition with common symptoms is not new [8] and has generated considerable debate as to whether depression inventories which include somatic symptoms should be used, modified or abandoned in populations with chronic physical conditions [9–12]. Despite the number of depression inventories used to assess people with MS and studies validating their use, there remains an evidence deficit in this area with only one pre-existing systematic review of such measures [13]. Minden and colleagues [13] reviewed measures used to assess and manage a wide range of psychiatric disorders in PwMS of which depression was just one, and sought to answer a number of clinical questions. The review had broad scope, did not assess the methodological quality of selected studies, and missed a large number of validation studies associated with the use of generic depression inventories in people with MS. Research studies can provide biased results if they lack methodological rigour [14]. To assess the report of research study accurately, readers need transparent information on its methods and results. This can be hampered if report authors fail to provide complete and clear descriptions of key study information [15]. For this reason, the COSMIN group have developed a critical appraisal checklist, which provides standards for the evaluation of the methodological quality of instrument validation studies [16]. The purpose of the present study was to review the evidence for the validity and reliability of self-report depression inventories in PwMS, in line with the COSMIN standards, with the aim of providing clinicians and researchers with a rational basis for choice.

## Methods

### Protocol and registration

A protocol for this systematic review is available on the PROSPERO database [17].

### Eligibility criteria

Eligible studies recruited people with MS, with no restrictions on duration or course of disease. Studies with comparison groups, for instance people with other physical or psychological conditions, were also included in the review. We included any study evaluating the validity or reliability of self-report instruments designed to assess depression. Studies simply assessing levels of depression, rather than the validity or reliability of measures, were not included. Studies focusing on the validation or reliability of neuropsychological tests of cognitive function or assessing general mental health or health-related quality of life, as opposed to depression, were excluded. We included peer reviewed research journal articles with primary research data. Commentaries, letters, dissertations and editorial papers were excluded. There was no restriction placed on publication date. Due to time and resource constraints, only English language papers were included.

### Information sources

We searched electronic bibliographic databases for published work as well as the reference lists of primary studies included in the review and of relevant, previously published reviews. We searched MEDLINE (from 1946 to 21.07.2014) and PsycINFO (from 1806 to July Week 3 2014) through OVID. Reference lists of eligible studies were checked and further relevant citations, not picked up through the searches of bibliographic databases, were included, along with serendipitous finds. No contact was made with study authors to retrieve unpublished information.

### Search

The search strategy included synonyms for the following terms: multiple sclerosis, depression assessment, validity and reliability. MeSH terms, exploding terms and other search devices relevant to each database were used to create a more efficient search. The full electronic search strategy for both databases is available on the PROSPERO database [17].

### Study selection

Two researchers (DK and either RW or DB) independently screened titles and abstracts for eligibility; differences were resolved by discussion with DH (5 papers [18–22]). The full papers of eligible titles and abstracts were retrieved and further reviewed for eligibility in the same way.

### Data collection process and data items

A standardised data extraction form was used, which included details about the study (authors, year, country), the samples (size, diagnoses, method of recruitment, baseline demographic characteristics), and types of validity or reliability assessed, as defined by the COnsensus-based Standards for the selection of health status Measurement INstruments checklist (COSMIN) checklist (e.g. internal consistency, reliability, measurement error, criterion validity, structural validity, content validity, cross-cultural validity) [23]. Outcome data were extracted as reported. One member of the review team (RW) extracted data, a second (DK) independently checked the extraction and a third (DH) checked a proportion of the extraction from the two members. When working through the COSMIN checklist, any discrepancies between the data extracted from the papers and the definition of the checklist were discussed within the team. For example, in one of the papers the authours [24] did not report that they were investigating construct validity, but reported the values for discriminant and convergent validity. Within the COSMIN checklist, construct validity includes aspects of structural validity, hypothesis testing and cross-cultural validity. In that particular case we reported the discriminant and convergent validity according to the COSMIN checklist and therefore were reported under 'Hypotheses Testing'.

### Risk of bias in individual studies

With a number of studies using different cut points in the evaluation of continuous variables we considered meta-analysis inappropriate [25]. As no statistical synthesis was planned, quality assessment was conducted for the purposes of describing the conduct of the included studies. DK assessed the included studies, unblinded, for generic dimensions of methodological quality defined by the COSMIN [23]. DH checked a 20 % random sample of ratings, which included all the poor rated papers and a random sample of the remaining papers and disagreements were resolved by discussion.

### Summary measures

We extracted the summary statistics, where reported, for domains, measurement properties, and their different aspects (see COSMIN taxonomy for further detail [16]).

For the reliability (extended definition) domain, we extracted: (1) inter-item correlations for the measurement property of internal consistency; item-subscale correlations, item-total correlations and Cronbach’s alpha (to assess internal consistency for the correlation between items) [26], with alpha scores of 0.70 and above acceptable by convention [27]; (2) Intraclass correlation coefficients (ICC) (with scores of 0.4–0.75 indicating fair to good reliability and higher scores excellent) [28] or Pearson or Spearman correlation coefficients (with scores of >0.7 considered acceptable [27]) for the measurement property of test-retest reliability; (3) standard error of measure (or statistics from which it could be derived) for the measurement property of measurement error.

For the validity domain, we extracted three categories of data. First, *p* values from *F*- or *t*-tests (with values less than 0.05 considered statistically significant), Cohen’s kappa (with values of >0.6 considered good agreement) [29], Spearman’s correlations (with scores of >0.7 considered acceptable [27]) and qualitative information on content validity. Second, narrative reports of factor analysis and uni-dimensionality of the items and, for confirmatory factor analysis (CFA), comparative fit index (CFI) and Tucker-Lewis Index (TLI) values ranging from 0 to 1, with a value ≥ 0.9 generally considered to indicate acceptable model fit, and the root mean square error of approximation (RMSEA) values of < .08 considered to indicate poor fit [30] (structural validity aspect), Pearson’s correlations and narrative information about convergent validity (hypotheses testing aspect), confirmatory factor analysis and narrative information on translation (cross-cultural validity aspect) for the measurement property of construct validity. Third, we extracted correlations (*r* statistics and p values), percentages, area under the curve (AUC), positive and negative predictive values (PPVs and NPVs) for continuous scores, or sensitivity and specificity at specified cut-points for dichotomous scores, for the measurement property of criterion validity.

## Results

### Study selection

Following the PRISMA reporting guidelines, the database searches retrieved 465 records of which 433 related to English language articles (Appendix 1). Five additional records were found through other sources; another review [13] and through the reference list of included papers [20–22, 31, 32]. After the elimination of duplicates there were 389 unique citations to screen of which 267 were excluded at the title stage and a further 82 at the abstract stage. Forty full text articles were retrieved for detailed assessment of eligibility, of these; a further 19 were excluded (see Appendix 2 for detailed reasons). Twenty-one studies (*N* = 5,991 people with MS) were included in the review (Appendix 3).

### Study characteristics

Included studies were published between 1995 and 2014 by teams based in the USA (*n* = 11 [7, 24, 33–41]), Canada (*n* = 6 [42–47]), the UK [31], France [48], Italy [49], and Estonia [50]. Only one study [49] examined the cross culture validity of the questionnaire in Italy. The median number of people with MS in the studies was 148 (Range = 42–1,717).

The median of the mean ages of people with MS reported in 18 out of 21 studies (Appendix 3, data missing from three studies [34, 42, 45]) was 45.79 years (range: 34.40–52.90). The median percentage of women reported in 19/21 MS cohorts (data missing from two studies [35, 42]) was 73.90 % (range: 63.00–83.30 %). The median of the mean of MS diagnosis in years reported in 11 out of 21 studies was 11.08 years (range: 6.6 - 19).

The included studies evaluated the reliability and/or validity of: the Beck Depression Inventory (BDI; *n* = 4 [7, 24, 39, 47]) or BDI-II (*n* = 1 [24]) or short-form variants the Fast Screen (BDI-FS) (*n* = 1 [36]) and ‘modified’ (mBDI) (*n* = 1 [41]); Center for Epidemiologic Studies Depression Scale (CES-D; *n* = 4 [43–45, 48]); Chicago Multiscale Depression Inventory (CMDI/MDI; *n* = 4 [35, 37, 40, 49]); Hospital Anxiety and Depression Scale (HADS; *n* = 2 [31, 42]); Patient Health Questionnaire (PHQ-9; *n* = 2 [33, 46]); CESD-10 (*n* = 1 [33]); Patient Reported Outcome Measurement Information System Depression 8-item bank (PROMIS-D-8; *n* = 1 [33]); Yale Single Question (YSQ; *n* = 1 [34]); a two-item measure of depression (‘During the past two weeks, have you often been bothered by feeling down, depressed, or hopeless?’ and ‘During the past two weeks, have you often been bothered by little interest or pleasure in doing things?’) (*n* = 1 [38]); and a one-item single question (‘Are you depressed?’) (*n* = 1 [50]). For the purposes of reporting we treat the BDI and BDI-II as one, but short-form versions (BDI-FS, mBDI) as separate entities. All the measures identified in this review are free to use with the exception of the BDI and HADS where there are costs associated with their use (Table 1).

**Table 1:** Reliability (Internal Consistency, Reliability & Measurement Error) Findings Based on Each Measure

Authors (Date)	Measure	Reliability (Cronbach’s Alpha)	Internal consistency (Inter-Item Correlations)	Test-test Reliability	Measurement error
Moran & Mohr (2005) [39]	BDI				BDI SEM = 3.26; HRSD SEM = 3.20
Aikens et al. (1999) [24]	BDI-II	Full BDI *α* = .86, Cognitive / Affective *α* = .85 Somatic *α* = .64 BDI-18 *α* = .84			
Amtmann et al. (2014) [33]	PHQ-9; CESD-10; PROMIS-D-8		PHQ-9 = .35-.67; CESD-10 = .33-.67; PROMIS-D-8 = .75-.84		
Sjonnesen et al. (2012) [46]	PHQ-9	*α* = .82	Item-total correlations (PwMS): anhedonia (.71); depressed mood (.65); fatigue (.57) and concentration (.55).		
Chang et al. (2003) [37]	CMDI	Sleep Disturbance *α* = .64 Cognitive Inefficiency *α* = .86 Fatigue *α* = .94 Evaluative *α* = .82 Mood *α* = .95			
Solari et al. (2003) [49]	CMDI	MS Vegetative *α* = .88 Evaluative *α* = .7 Mood *α* = .93	Item-subscale: mood = .58-.80, evaluative = .37-.70, vegetative = .32-.57	ICC = 0.78 (95 % CI 0.62–0.89); Subscales range 0.71–0.79 (2 weeks)	
Patten et al. (2010) [45]	CES-D			*r =* .69 over 1 year; *r* = .70 over 2 years; *r* = .65 over 3 years; *r* = .73 year 1-year 2; *r* = .73 year 2-year 3	
Verdier-Taillefer et al. (2001) [48]	CES-D	*α* = .90			

Note: HRSD = Hamilton Rating Scale for Depression

The included studies evaluated: construct validity (*n* = 16 [7, 24, 31, 35–41, 43, 44, 46–49]); criterion validity (*n* = 9 [31, 34, 36, 38, 42, 43, 47, 49, 50]); internal reliability (n = 5 [24, 37, 46, 48, 49]); content validity (*n* = 4 [37, 46, 48, 49]); test-retest reliability (*n* = 2 [45, 49]); discriminant/convergent validity [33]; evaluation of dimensionality [33]; and inter-item reliability [33] (Table 2).

**Table 2:** Structural and Content Validity Findings by Measure

Authors (Date)	Measure	Structural Validity (Factor Analysis)	Content Validity Item Inclusion
Mohr et al. (1997) [70]^a^	BDI		Fatigue, work difficulty, and concerns about health (*p*s < .001) within MS population
Moran & Mohr (2005) [39]	BDI		All items showed significant reductions following treatment (*p* < .05)
Aikens et al. (1999) [24]	BDI-II		MS had higher scores than healthy controls on work difficulty, *F* = 8.05, *p* < .001 and sexual disinterest, *F* = 9.99, *p* < .005.
Strober & Arnett (2010) [41]	mBDI		MS-NON-DEP: fatigue, indecision, loss of libido, work difficulty, irritability, loss of interest, crying, dissatisfaction, self-criticism. DEP-PwMS: Irritability, loss of interest, crying, dissatisfaction, self-criticism, sadness, pessimism, failure, guilt, appetite, disappointment, weight loss. MS (more severe in DEP-MS) or related to depressive symptoms: irritability, loss of interest, crying, dissatisfaction, self-criticism.
Amtmann et al. (2014) [33]	PHQ-9; CESD-10; PROMIS-D-8	Fit indices from a one-factor CFA were acceptable. CFI for all models ≥ of 0.95. TLIs for PHQ-9 and CESD-10 < 0.95 (0.94 and 0.93, respectively). TLI > 0.95 for PROMIS-D-8. RMSEAs > .05 for any of the measures.	
Sjonnesen et al. (2012) [46]	PHQ-9		Exclusion of fatigue and concentration items no change in prevalence estimates (*p* > .05). Fatigue item contributed the most to the total score in both groups (MS 35.1 % (95%CI 30.9–39.3), control 34.8 % (95%CI 33.5–36.1). Item contribution for fatigue and concentration items between groups (*p >* .05). Anhedonia was lower in MS than controls (OR 0.47, *p* = .03), PwMS positively endorsed guilt (OR 2.17, *p* = .03) and fatigue (OR 1.51, *p* = .05); frequency of endorsement between groups (*p* > .05)
Patten et al. (2005) [44]	CES-D		Agreement between full and modified scales, *r* = .99, 1.00, .98; *k* = .93, .96, .90. Prevalence for full scale (>15) was 32.8 % (95%CI 18.9–36.8), exc. fatigue 30.0 % (95%CI 26.3–34.0), exc. cognitive 31.0 % (95%CI 27.3–35.0), exc. both 30.4 % (95%CI 26.6–24.4).
Verdier-Taillefer et al. (2001) [48]	CES-D	Factor 1 = depressed affect; Factor 4 = interpersonal relationships. Factor 2 = positive affect items (MS group), but contained somatic complaints (other groups); Factor 3 = somatic complaints (MS group) but positive affect (other groups)	
Chang et al. (2003) [37]	CMDI	CFA confirmed the subscale/factor structure	Vegetative subscale showed misfitting in PwMS. ‘Fatigue’ and ‘useless’ items were endorsed more by MS than controls.
Mohr et al. (2007) [38]	Two-item measure		The sensitivity and NPV of using either question alone significantly better than either question alone; the sensitivity of both questions was significantly lower than other methods

Note: Studies that are not included in the table either did not assess content validity or did not report it. ^a^ ANOVAS but does not report *f* values. DEP = Depressed

The median sensitivity score of the measures used, reported in six criterion validity studies (Table 4), was 73.15 % (range: 25.00–90.00 %) [31, 34, 38, 42, 47, 50]. The median specificity score of the measures used, reported in six studies, was 87.30 % (range: 46.00–98.00 %) [31, 34, 38, 42, 47, 50]. The median PPV score of the measures used, reported in seven studies, was 90.00 % (range: 54.00–96.80 %) [34, 38, 42, 43, 47, 49, 50]. The median NPV score of the measures used, reported in three studies, was 84.00 % (range: 78.50–85.00 %) [34, 38, 47] (Tables 3 and 4).

**Table 3:** Hypotheses Testing Findings by Measure

Authors (Date)	Measure	Discriminant	Convergent
Aikens et al. (1999) [24]	BDI-II	BDI totals differed by group, *F* = 30.99, *p* < .001; controls less depressed than MS, *p* < .05, and MS less depressed than chronic pain patients, *F* = 12.9 *p* < .001. On the somatic subscale, MS scored higher than all other groups (*p*s < .001), except diabetes patients (ns)	BDI totals similar for MS and diabetes *F* = 3.84, *p* = ns
Moran and Mohr (2005) [39]	BDI	BDI total score reduced from 23.7 (*SD* = 6.9) to 10.5 (*SD* = 6.5) following treatment, *t* = 10.91, *p* < .001	
Sullivan et al. (1995) [47]	BDI	Participants with major depression scored significantly higher on negative attitude towards self and performance components, *F*(2,41) = 3.98, *p* < .05, *F*(2,41) = 3.42, p < .05, but not on the somatic component, *F*(2,41) = 1.30, *p* = ns.	
Benedict et al. (2003) [36]	BDI-FS	Groups differed in scores between treated and untreated groups (anti-depressants), *F* = 13.26, *p* < .01, persisting after controlling for physical disability. Treatment effect size was largest for BDI-FS than other measures (*d* = 1.5).	
Strober and Arnett (2010) [41]	mBDI	PwMS (DEP, NON-DEP) endorsed fatigue (*p* < .001), work difficulty (*p* < .001), indecision (*p* < .01), irritability (*p* < .01), loss of libido (*p* < .05), loss of interest (*p* < .01), crying (*p* < .05), dissatisfaction (*p* < .05 and self-criticism (*p* < .05) on the BDI	BDI and depression proneness rating scale, *r* = .52, *p* < .01; BDI and CMDI, *r* = .76, *p* < .01
Amtmann et al. (2014) [33]	PHQ-9; CESD-10; PROMIS-D-8	PHQ-9 with CESD-10 (.85); PROMIS-D-8 (.73). CESD-10 with PROMIS-D-8 (.80).	Fatigue scores correlations: PHQ 9 = .73; CESD-10 = .71; PROMIS-D-8 = .55. Sleep disturbance: PHQ-9 = .57; CESD-10 = .56; PROMIS-D-8 = .39. Pain interference: PHQ-9 = .60; CESD-10 = .55; PROMIS-D-8 = .47.
Pandya et al. (2005) [43]^a^	CES-D	MS with a major depressive disorder diagnosis had CES-D score (36.5) than those who did not (26.0), Mann-Whitney = 12.1, df = 1, *p* < .001	
Patten et al. (2005) [44]	CES-D	CES-D scores did not correlate as highly with physical QoL, as with mental QoL, Spearman's *r* = -.64. CES-D scores and the physical, Spearman's *r* = .61, or the cognitive, Spearman's *r* = .66, subscales of the fatigue impact scale.	CES-D scores correlated highly with mental QoL, Spearman's *r* = -.80. CES-D scores and the Fatigue Impact Scale social subscale, Spearman's *r* = .74.
Verdier-Taillefer et al. (2001) [48]	CES-D	PwMS endorsed more depressed affect and fatigue items than GP patients; MS and GP patients endorsed more depressive symptoms than healthy workers (*ps* < .05)	
Beeney and Arnett (2008) [35]	CMDI	Physical disability was correlated with vegetative subscale, *r*(94) = .36, *p* < .05; mood, *r*(94) = .16, *p* = ns, or evaluative, *r*(94) = .20, *p* = ns, subscales.	History of depression correlated with CMDI-Mood, *r*(94) = .38, *p* < .001; CMDI-Evaluative, *r*(94) = .31, *p* < .001; CMDI-Vegetative *r*(94) = .10, *p* = ns. Depression proneness correlated with mood, *r*(94) = .53, *p* < .001; evaluative, *r*(94) = .48, *p* < .001, subscales; vegetative subscale, *r*(94) = .15, *p* = ns.
Chang et al. (2003) [37]	CMDI	PwMS scored higher on all dimensions than controls (CMDI-Mood *t* = 6.10, *p* < .001, CMDI-Evaluative *t* = 6.61, *p* < .001, CMDI-Vegetative *t* = 8.04, *p* < .001).	
Nyenhuis et al (1995) [40]	MDI	PwMS endorsed more vegetative symptoms than mood and evaluative, than the depressed group, controlling for total BDI score *F*(2,322) = 5.7, *p* < .01; PwMS endorsed less mood items than the depressed group, *t*(161) = 2.5, *p* < .05, with no difference in evaluative, *t*(163) = 0.34, *p* > .10, or vegetative, *t*(163) = -0.58, *p* > .10 items.	BDI (30.5 %) estimated depression significantly greater than MDI-mood (17.7 %), *p* < .05; MDI-Vegetative (34.6 %) estimated significantly higher prevalence than MDI-Evaluative (22.2 %), *p* < .05, and MDI-Mood, *p* < .01; MDI-Mood estimated lower prevalence than MDI-Total (26.6 %), *p* < .05.
Solari et al. (2003) [49]	CMDI	Significant differences in all subscale scores, *p* < .001. Adjusted odds ratios for MS vs. healthy controls for depression was 2.72 (95%CI 1.14–6.50, *p* = .02) total scale; 2.79 (95 %CI 1.19–6.54, *p* = .02); mood scale, 2.00 (95%CI 0.88–4.56, *p* = .10); evaluative scale; 6.49 (95%CI 1.92–21.93, *p* = .001) vegetative scale.	
Nicholl et al. (2001) [31]	HADS-D		HADS-D correlated with both the GHQ-12 (*r* = .49, *p* < .01), and the GHQ-28 (*r* = .48). Kappa between HADS-D and BDI 0.12, p = .22.

Note: studies that are not included in the table either did not assess construct validity or did not report it. ^a^Paper talks about Mann-Whitney chi square – test does not exist. QoL = Quality of Life; GHQ = General Health Questionnaire

**Table 4:** Criterion Validity Findings by Measure

Authors (Date)	Measure	Criterion Measure	Correlation	Sensitivity	Specificity	ROC Curve Analysis	PPV	NPV
Benedict et al (2003) [36]	BDI-FS	BDI, CES-D, neuropsychiatric inventory (NPI)	BDI *r* = .85, *p* < .001; CES-D *r* = .86, *p* < .001; NPI subscales range *r* = .32-.50, *ps* < .05					
Sullivan et al. (1995) [47]	BDI	Structured clinical interview (DSM)		88 % (≥9), 71 % (≥13)	46 % (≥9), 79 % (≥13)		54 % (≥9), 70 % (≥13)	84 % (≥9), 79 % (≥13)
Honarmand and Feinstein (2009) [42]	HADS-D (≥8)	SCID-I (DSM)		90 %, (95%CI 73–98)* 86.7 % (95%CI 68–96)* fatigue item excluded (≥6)	87.3 %, (95%CI 81–92)* 86.7 % (95%CI 80–92)* fatigue item excluded (≥6)	AUC = 0.94, 0.93 fatigue item excluded (≥6)	90.6 %, 90.6 % fatigue item excluded (≥6)	
Nicholl et al. (2001) [31]	HADS-D	BDI (Predictive)	*r* = .58, *p* < .01	25 % - When using cut-off of 5/6 (identified by ROC curve), 75 %	86 % - When using cut-off of 5/6 (identified by ROC curve), 69 %	Identified the optimum cut-off of 5/6 when compared to the BDI.		
Avasarala et al. (2003) [34]	YSQ	BDI (Cut-off of ≥13)		65.3 % (95%CI 50–78)	87.3 % (95%CI 77–94)		78.0 % (95%CI 62–89)	78.5 % (95%CI 68–87)
Mohr et al. (2007) [38]	Two-item measure	SCID (DSM)		Both questions: 51 % (95%CI 38–63), either: 99 % (95%CI 91–100), 1 only: 75 % (95%CI 62–84), 2 only: 75 % (95%CI 62–84)	Both: 98 % (95%CI 94–99), either: 87 % (95%CI 81–91), 1 only: 94 % (95%CI 89–97), 2 only: 94 % (95%CI 89–97)		Both: 90 % (95%CI 74–97), either: 72 % (95%CI 61–80), 1 only: 73 % (95%CI 61–83), 2 only: 81 % (95%CI 68–89)	Both: 85 % (95%CI 80–89), either: 99 % (95%CI 96–100), 1 only: 91 % (95%CI 86–95), 2 only: 91 % (95%CI 86–95)
Pandya et al. (2005) [43]	CES-D	DSM-IV psychiatric interview					59.6 % (95%CI 45.0–74.1) for major depression; 74.5 % (95%CI 61.5–87.4) for any depressive disorder.	
Solari et al. (2003) [49]	CMDI	Previously diagnosed according to DSM-IV					Total scale: 96.8 %; mood: 96.9 %; evaluative and vegetative: 75.0 %	
Vahter et al (2007) [50]	One-item measure	BDI (>10 cut-off) and structured clinical interview		81%^**a**^	89%^**a**^		93.5%^**a**^	

Note: Studies that are not included in the table either did not assess criterion or did not report it.^a^Extracted from Manoj and Sivan (2007) [52] ^*^Calculated by EL. SCID = Structured Clinical Interview for DSM Disorders; ROC = Receiver operating characteristic

### Risk of bias within studies (COSMIN)

For the BDI / BDI-II and brief versions (BDI-FS, mBDI; see Table 5), the quality of information was rated: fair for overall reliability (*n* = 2 [24, 39]); fair (*n* = 2 [36, 47]) or excellent (*n* = 4 [7, 24, 39, 41]) for content validity; fair for hypothesis testing (*n* = 7 [7, 24, 36, 39–41, 47]); fair for criterion validity (*n* = 2 [36, 47]); fair for responsiveness (*n* = 1 [39]). No papers measured the structural validity or cross-cultural validity of the BDI. For the BDI-FS and the mBDI, no papers assessed reliability, structural validity, cross-cultural validity, or responsiveness; and, for the mBDI no paper assessed criterion validity.

**Table 5:** Methodological Quality of Each Article per Measurement Property and Instrument According to COSMIN Checklist

PAPERS	Measure	Internal Consistency	Reliability	Measurement Error	Content Validity (Face Validity Incl.)	Structural Validity	Hypotheses testing	Cross Cultural Validity	Criterion Validity	Responsiveness
Mohr et al. (1997) [70]	BDI	-	-	-	Excellent	-	Fair	-	-	-
Moran & Mohr (2005) [39]	BDI	-	-	Fair	Excellent	-	Fair	-	-	Fair
Sullivan et al. (1995) [47]	BDI	-	-	-	Fair	-	Fair	-	Fair	-
Aikens et al. (1999) [24]	BDI-II	Fair	-	-	Excellent	-	Fair	-	-	-
Benedict et al. (2003) [36]	BDI-FS	-	-	-	Fair	-	Fair	-	Fair	-
Strober & Arnett (2010) [41]	mBDI	-	-	-	Excellent	-	Fair	-	-	-
Pandya et al. (2005) [43]	CES-D	-	-	-	-	-	Fair	-	Poor^d^	-
Patten et al. (2005) [44]	CES-D	-	-	-	Excellent	-	Fair	-	-	-
Patten et al. (2010) [45]	CES-D	-	Fair	Fair	-	-	Good	-	-	Fair
Verdier-Taillefer et al. (2001) [48]	CES-D	Good	-	-	Excellent	Good	Good	-	-	-
Amtmann et al. (2014) [33]	CESD-10; PHQ-9; PROMIS-D-8	Poor^a^	-	-	Fair	Fair	Fair	-	-	-
Sjonnesen et al. (2012) [46]	PHQ-9	Poor^b^	-	-	Fair	-	Fair	-	-	-
Beeney & Arnett (2008) [35]	CMDI	-	-	-	Excellent	-	Fair	-	-	Fair
Chang et al. (2003) [37]	CMDI	Fair	-	-	Excellent	Fair	Fair	-	-	-
Nyenhuis et al. (1995) [40]	MDI	-	-	-	Fair	-	Fair	-	-	-
Solari et al. (2003) [49]	CMDI	Fair	Fair	-	Excellent	-	Fair	Poor^c^	-	-
Honarmand & Feinstein (2009) [42]	HADS	-	-	-	-	-	Fair	-	Fair	-
Nicholl et al. (2001) [31]	HADS	-	-	-	-	-	Good	-	Good	-
Avasarala et al. (2003) [34]	YSQ	-	-	-	-	-	Fair	-	Fair	-
Mohr et al. (2007) [38]	Two-item measure	-	-	-	Excellent	-	Fair	-	Fair	-
Vahter et al (2007) [50]	One-item measure	-	-	-	-	-	Fair	-	Fair	-

Notes: ^a^ This paper was rated poor due to not calculating internal consistency for each subscale separately. ^b^ This paper was evaluated poor for not assessing the unidimensionality of the measure, although we understand that might not have been the purpose of the study. ^c^ Multiple group Confirmatory Factor Analysis or Differential Item Function not performed/assessed. ^d^ No correlations or AUC calculated

For the CES-D, the quality of information was rated: poor (*n* = 1 [33]) or fair (*n* = 1 [45]) for overall reliability; fair (*n* = 1 [33]) or excellent (*n* = 2 [44, 48]) for content validity; fair (*n* = 1 [33]) to good (*n* = 1 [48]) for structural validity; fair (*n* = 3 [33, 43, 44]) to good (*n* = 2 [45, 48]) for hypothesis testing; poor (*n* = 1 [43]) to fair (*n* = 1 [33]) for criterion validity; fair for responsiveness (*n* = 1 [45]). No paper assessed cross-cultural validity.

For the CMDI, the quality of information was rated: fair for overall reliability (*n* = 2 [37, 49]); excellent for content validity (*n* = 3 [35, 37, 49]); fair for structural validity (*n* = 1 [37]); fair for hypothesis testing (*n* = 3 [35, 37, 49]); poor for cross-cultural validity (*n* = 1 [49]); fair for responsiveness (*n* = 1 [35]). No paper assessed criterion validity.

For the PHQ-9, the quality of information was rated: poor for overall reliability (*n* = 2 [33, 46]); fair for content validity (*n* = 2 [33, 46]); fair for structural validity (*n* = 1 [33]); fair for hypothesis testing (*n* = 2 [33, 46]); fair for criterion validity (*n* = 1 [33]). No paper assessed cross-cultural validity or responsiveness.

For the two-item measure, the quality for the information was rated: excellent for content validity; fair for hypothesis testing and fair for criterion validity [38]; no paper assessed overall reliability, structural and cross-cultural validity or responsiveness.

For the HADS, the quality of information was rated: fair (*n* = 1 [42]) to good (*n* = 1 [31]) for hypothesis testing; and, fair (*n* = 1 [42]) to good (*n* = 1 [31]) for criterion validity. For the YSQ, the quality for the information was rated: fair (*n* = 1 [34]) for hypothesis testing; and, fair (*n* = 1 [34]) for criterion validity. For the one-item measure, the quality for the information was rated: fair (*n* = 1 [50]) for hypothesis testing; and, fair (*n* = 1 [50]) for criterion validity. No paper assessed reliability, content validity, structural validity, cross-cultural validity or responsiveness of the HADS, the YSQ or the one-item measure.

### Synthesis of results

#### BDI

For the reliability of the BDI (*n* = 2), internal consistency was generally good (*α* = 0.86 for the full BDI-II [24]) with a standard error of measurement (SEM) of 3.26 for test-retest reliability [39], but only acceptable for the somatic symptom cluster (*α* = 0.64 [24]). In studies of content validity, people with MS and major depressive disorder (MDD) had higher scores than controls (on the BDI-II [24]) and were responsive to treatment [39]; one study reported that the BDI lacked face validity for people with MS [7], but this was contradicted by later studies [24, 39]. Studies tested hypotheses related to somatic symptoms common to MS and depression (*n* = 3 [7, 24, 39]) and the general utility of the instrument in people with MS (*n* = 1 [47]). The findings for the studies were conflicted with one study providing evidence that the BDI was sensitive enough to show difference in treatment scores [39] and with other studies providing evidence that the BDI is confounded by MS-related symptoms [7]. All researchers called for more research on the overlap of MS and depression symptoms and the utility of depression measures for PwMS. For criterion validity (Table 4), using DSM-diagnosed MDD as a gold standard, the BDI demonstrated 71 sensitivity and 79 % specificity for a cut point of 13 points (mild depression [47]).

In studies of content validity, non-depressed PwMS scored highly on a number of symptoms of depression measured by the mBDI (Table 2 [41]), and face validity was investigated for both the mBDI [41] and the BDI-FS [36]. The BDI-FS was found to be more sensitive to treatment effects than the HDRS (or HADS), whereas Strober and Arnett [41], suggested a sort of hierarchy in assessing depression in PwMS. Strober and Arnett [41] suggested that the following (‘branch’) symptoms sadness, pessimism, sense of failure, disappointment and changes in appetite and/or weight were indicative of depression in MS. On the other hand they suggested that the following (‘trunk’) symptoms, fatigue, work difficulty, indecision, irritability, loss of interest, loss of libido, crying, dissatisfaction, self-criticism, were common in MS but were indicative of depression if the symptoms were disproportionate to the medical illness [41]. The hypotheses tested related to the general validity of the BDI-FS [36] and somatic symptoms common to MS and depression for the mBDI [41]. The BDI-FS demonstrated moderate correlations (internal consistency) with the neuropsychiatric inventory and strong correlations with the BDI and CES-D [36]. The BDI-FS was strongly correlated (criterion validity) with the BDI-II (*r* = 0.85) and CES-D (*r* = 0.86 [36]).

#### CES-D

For the reliability of the CES-D (*n* = 3), internal consistency was excellent (*α* = 0.90 [48]), but inter-item correlations were poor to acceptable (0.33–0.67 [33]) and test-retest reliability ranged from moderate (*r =* 0.65) to strong (*r =* 0.73) over different time intervals [45]. In studies of content validity (*n* = 3 [33, 44, 48]), there was strong agreement between the results of tests with and without fatigue and cognition questions (*r =* 0.98–1.00; *k =* 0.90–0.96 [44]) suggesting that the instruments were not contaminated by the presence of somatic symptoms common to MS and depression. The issue of contamination is addressed fully in the Discussion section. An investigation of face validity found that CES-D captured a broad range of depression (including mildly, moderate and severely depressed); however there were conflicting results on the measure's structure for the MS population [33, 44, 48]. For structural validity (*n* = 2 [33, 48]) the fit indices for a one-factor CFA were acceptable, with CFI of ≥0.95, TLI <0.95 and a RMSEA which did not meet the recommended level [33]. In an exploratory factor analysis (EFA), four factors (depressed affect, positive affect, somatic complaints and interpersonal relationships) explained more than 50 % of the variance in test scores between MS and non-MS populations indicating good structural validity [48]. Studies tested hypotheses related to the utility or predictive value [43, 44], longitudinal utility [45], and the general reliability and validity of the scale [48]. Using the DSM-diagnosed MDD as a gold standard (criterion validity), the PPV of the CES-D was 59.6 % [43]; the CES-D was strongly correlated with the PHQ-9 (*r* = 0.85 [33]). For responsiveness, insufficient data were available to calculate the SEM [45].

#### CMDI

For the reliability of the CMDI (*n* = 2 [37, 49]), no overall value was available for internal consistency, but internal consistency of the subscales was generally acceptable to excellent (*α* = 0.64–0.95 [37, 49]), the low value being for sleep disturbance [37]. Researchers observed intra-class correlations of 0.71 to 0.79 over two weeks for test-retest reliability [37]; inter-item correlations were moderate-to-strong for mood (0.58 to 0.80) and evaluative (0.37 to 0.70) but moderate for vegetative (0.32 to 0.57) subscales [49]. In studies of content validity (*n* = 4 [35, 37, 40, 49]), the vegetative subscale was shown to be misfitting (unweighted item fit mean square <0.7 or >1.3 [51]) with the MS participants more likely to endorse the fatigue and feeling useless items than controls [37]; investigation of face validity found that the CMDI had a 'consistent overlap' with the MS symptoms [35, 40, 49]. Studies tested hypotheses related to somatic symptoms common to MS and depression (*n* = 2 [35, 40]), the general reliability and validity of the instrument in people with MS (*n* = 1 [37]) and cross-cultural validation of the instrument (*n* = 1 [49]). Overall, the papers consistently found that there should be a distinction between depression and MS symptoms and that the CMDI provided a good measure for that. Regarding structural validity (*n* = 1 [37]) a five-factor CFA found an excellent fit between the proposed model and data, with CFI of 0.93, the normed fit index (NFI) of 0.90, the non-normed fit index (NNFI) of 0.92. For responsiveness there was insufficient data available in any study to calculate the SEM [35]. There were no studies which assessed criterion validity.

#### HADS

For the HADS, studies tested hypotheses related to the general utility of the measure [31, 42] and found it to be a useful screening instrument with high criterion-related validity for depression. Using the DSM-diagnosed MDD as a gold standard (criterion validity), the sensitivity and specificity of the HADS were 90 and 87.3 % for the whole scale or, 86.7 % for both when fatigue items were excluded [42]. Using the BDI as a gold standard, the HADS had a sensitivity and specificity of 75 and 69 % respectively, and the two were moderately correlated (*r* = 0.58 [31]). No study evaluated the reliability, the content validity, the structural validity or cross cultural validity of the HADS.

#### PHQ-9

Regarding the reliability of the PHQ-9 (*n* = 2), internal consistency was good (α = 0.82 [46]) with inter-item correlations of 0.35–0.67 (moderate to acceptable [33]); item-total correlations were good for anhedonia (0.71), acceptable for depressed mood (0.65), and moderate for fatigue (0.57) and concentration (0.55) subscales [46]. Fatigue items made the highest contribution to the total score in all participants, with no significant differences between MS and control participants (content validity [46]). For structural validity (*n* = 1 [33]) the fit indices for a one-factor CFA were acceptable, with a CFI of >0.95, TLI <0.95 and the RMSEA did not meet the recommended level (>0.05). Studies tested hypotheses (*n* = 2) related to somatic symptoms [46] and the comparative psychometric properties of different scales [33]. One study tested hypotheses related to the convergent validity of PHQ-9, the CESD-10 and PROMIS-D-8 [33]. No study evaluated the criterion validity or cross cultural validity of the PHQ-9.

One study tested hypotheses related to the criterion validity of the YSQ. Using the BDI as a gold standard, the sensitivity and specificity were 65.3 (95 CI 50–78)% and 87.3 (95 % CI 77–94)% respectively [34]. No study evaluated the reliability, content validity, structural validity, cross cultural validity or responsiveness

## Discussion

### Principal Findings

In this section, we summarise the evidence for dimensions of validity across different instruments, referring in brackets to the number of participants and the COSMIN-rated quality of each study. The criterion validity of instruments, using DSM-diagnosed MDD as a gold standard, can be ranked in order of decreasing sensitivity and specificity, where reported, as follows. For sensitivity: (1) HADS (*n* = 140; Fair) 90 %; (2) the one-item measure (*n* = 134; Fair [50, 52]) 81 %; (3) BDI (*n* = 46; Fair [47]) 71 %; (4) the two-item measure (*n* = 260; Fair [38]) 51 %. For specificity: (1) the two-item measure (*n* = 260; Fair [38]) 98 %; (2) the one-item measure (*n* = 134; Fair [50, 52]) 89 %; (3) HADS (*n* = 140; Fair) 87 %; (4) BDI (*n* = 46; Fair [47]) 79 %. The structural validity of instruments, using a variety of methods can be ranked as follows. For CFA: (1) CMDI (*n* = 433; Fair [37]) excellent; (2=) CESD (*n* = 455; Fair [33]) acceptable; (2=) PHQ-9 (*n* = 455; Fair [33]) acceptable.

There was little to distinguish the three measures (CES-D, PHQ-9 and CMDI) in terms of their CFI scores or, for the CES-D and PHQ-9, their TLI or RMSEA scores (with the latter not meeting the recommended level in either case). The method of assessing content validity across studies varied widely, prohibiting any ranking of studies. In brief, the CES-D (three studies rated fair [33] or excellent [44, 48]) found no issue with the inclusion of somatic symptoms, whereas the BDI (6 studies rated as fair [36, 47] or excellent [7, 24, 39, 41]), and CMDI (3 studies rated as excellent [35, 37, 49]) identified some issues with the inclusion of somatic symptoms in at least one study [24].

The most sustained debate on content validity surrounded the BDI. While Mohr et al. (1997) [7], found that ‘fatigue’, ‘work difficulty’ and ‘concerns about health’ items were MS-confounded (disproportionately endorsed within PwMS) on the BDI, this was not confirmed by Aikens et al. [24] (using the BDI-II), or Moran and Mohr [39], who concluded all items were suitable for inclusion. Those studies which presented an overall score for internal consistency (reliability) can be ranked as follows: (1) CES-D (*n* = 857; Good [48]) *α* = 0.90; (2) BDI-II (*n* = 105; Fair [24]) *α* = 0.86; (3) PHQ-9 (*n* = 173; poor [46]) *α* = 0.82.

### Strengths and limitations

The current review both updates and overcomes a number of limitations of the earlier review by Minden and colleagues. First, our search strategy was considerably more sensitive, identifying 15 eligible studies which had been published at the time of the searches (August 2011) for the Minden article. Although our search strategy is limited in its use of bibliographic databases, we believe the associated risk of bias is low. When resources are constrained, the use of Medline and PsycINFO, along with checking reference lists, is justifiable in some topic areas [53]. In mental health research, each database performs well in the retrieval of mental health literature [54], and their use in combination is recommended [55]. On the other hand, CINAHL rarely retrieves unique references for most topic areas [56]. The exclusion of non-English language studies can have more impact on the comprehensiveness of a review, although there is no evidence that it causes systematic bias [57]. Empirical evidence of bias stemming from the omission of grey literature is available for systematic reviews of therapeutic interventions [58], but not from reviews of validation studies. The impact of this omission on our review and the one by Minden and colleagues is unknown, but future reviews might supplement searches of bibliographic databases with grey literature databases such as PsycEXTRA and the ProQuest Dissertations & Theses Database, in addition to Google searches.

Systematic reviews which critically appraise and compare the measurement properties of different instruments can provide a sound basis for the selection of instruments [59]. Unlike the Minden team, we took a formal approach to the assessment of study quality using the COSMIN checklist [23]. While some will find this helpful, the rating of validation studies can be argued to be subjective, especially on issues such as face validity. We were unable to undertake statistical synthesis due to the absence of confidence intervals associated with summary statistics in most reports. Our findings are also affected by outcome reporting bias, especially in studies evaluating reliability and criterion validity; for instance, of 10 studies evaluating criterion validity seven presented sensitivity and specificity, eight presented PPV and four presented NPV. Outcome reporting bias may present a similar threat to the credibility of overviews of validation studies as documented in systematic reviews of therapeutic interventions [60, 61], and psychometricians should consider following the Core Outcome Measures in Effectiveness Trials (COMET) initiative in the development of core outcome sets for the COSMIN domains [62].

### Key messages for people with MS, therapists, policy-makers

#### Item inclusion & symptom overlap

Concerns over item inclusion, given the overlap between MS and somatic depressive symptoms, were expressed by authors of studies included in this review, with self-report measures providing no opportunity to probe whether depressive symptoms are influenced by disease severity [63]. Despite such symptom overlap, the exclusion of items on this basis is questionable [64]. A suggested alternative is the use of different instruments, such as the BDI-FS, which is less reliant on the somatic symptoms that can confound assessment [36], or the BDI-II [65], which removes a number of items that might be MS related. Similarly, a different interpretation or analysis of standard BDI scores, such as the ‘trunk and branch’ model proposed by Strober and Arnett, separating out those symptoms common to MS, may help to overcome such issues of overlap [41]. An alternate approach would be to adopt more generic measures that assess extent to which physical and mental health problems impact on a person’s quality of life [66]. In this context, a lack of functioning or activity impairment is important to the extent to which it impacts on quality of life, rather than the severity of their symptoms or condition. This might involve the use of generic measure of health related quality of life, such SF-36, or measures that better reflect the impact of mental health problems identified in the literature [67].

#### Usefulness of measures

The one- or two-item measures [38, 50] may be preferable for use in people with MS (PwMS) as they are quick and easy to administer, potentially overcoming any difficulties arising from cognitive impairment [34]. Furthermore, they do not contain items relating to somatic symptoms and so may overcome the overlap issues identified above. However, they only offer an indication of the presence of depression and not its severity, which may be useful to clinicians in identifying cases but not to researchers in assessing treatment responsiveness, for example.

#### Assessment criterion

Researchers and clinicians should consider the utility of the measures discussed in relation to their ability to identify probable cases of depression. As such, when using self-report scales as a case finder, it is important to identify the optimum cut-off value [47]. This is difficult as studies often use different cut-off values to signify a case (somebody who, whilst not diagnosed, could benefit from talking therapy) and sub-standard criterion against which to assess the measure (e.g. the BDI rather than structured clinical interview). A robust criterion gives greater confidence around the recommendation of such cut-off values. This review suggests that the modification of the BDI, accounting for MS symptoms, may be an effective way to assess depression in this patient group [63], though further independent research is warranted.

#### Need for further research

High quality validation studies are currently unavailable and are needed on the following topics: the reliability of the two-item measure, HADS, YSQ, the one-item measure; the structural validity of the BDI, two-item measure, HADS, YSQ, the one-item measure; the cross-cultural validity of the BDI (and its short-form variants), CES-D, PHQ-9, CESD-10, PROMIS-D-8, two-item measure, HADS, YSQ, the one-item measure; the responsiveness of the two-item measure, HADS, YSQ, the one-item measure review.

Further evidence is needed for most measures in assessing their utility in this patient group, along with the appropriateness of some of the items they use. Whilst item exclusion may not be appropriate, altered scoring or interpretation may be necessary. More investigation of existing measures should seek to compare them against structured clinical interview, a gold standard measure, given the questions raised regarding the validity of the BDI [47]. Given the prevalence of BDI-based measures, further research should seek to validate the BDI-II, mBDI and BDI-FS in people with MS. Use of measures besides the BDI should be considered, such as the HADS and PHQ-9, given their apparent robustness to confounding from MS symptoms. Assessing the accepability of instruments to PwMS using qualitative methods, such as Think Aloud methodology, is an essential complement to quantitative evaluation [68, 69].

## Conclusions

All instruments identified in this review need further work on validation and reliability for use in people with MS. On the basis of the available evidence regarding these measures, researchers and practitioners are faced with trade-offs depending on their priorities. In addition, researchers conducting further studies need to pay special attention to the contamination of the depression inventory scores overlapping with the MS symptoms. However, it may be that a strategic re-evaluation is required in the approach to measuring depression in people with MS. Rather than researchers pursing a piecemeal approach to specific psychometric properties of a range of outcome measures, an alternative might be to move towards constructing and adopting Quality of Life measures that emphasise the fulfilment of a person’s needs rather than prioritising the severity of specific symptoms.

## Abbreviations

AUC, area under the curve; BDI / BDI-II, Beck Depression Inventory; BDI-FS, Beck Depression Inventory-Fast Screen; CES-D, Center for Epidemiologic Studies Depression Scale (identical to the CESD-10); CESD-10, Center for Epidemiologic Studies Depression Scale 10-item scale (identical to the CES-D); CFA, confirmatory factor analysis; CFI, comparative fit index; CI, confidence interval; CINAHL, Cumulative Index to Nursing and Allied Health Literature; CMDI / MDI, chicago multiscale depression inventory; COSMIN, COnsensus-based Standards for the selection of health status Measurement INstruments; DSM, Diagnostic and Statistical Manual of Mental Disorders; HADS, Hospital Anxiety and Depression Scale; ICC, Intraclass correlation coefficient; mBDI, Modified Beck Depression Inventory; MDD, major depressive disorder; MeSH, Medical Subject Headings; MS, multiple sclerosis; NPV, negative predictive value; PHQ-9, Patient Health Questionnaire; PPV, positive predictive value; PROMIS-D-8, Patient Reported Outcome Measurement Information System Depression 8-item bank; PROSPERO, International PROSPEctive Register Of systematic reviews; PwMS, people with multiple sclerosis; RMSEA, root mean square error of approximation; SEM, standard error of measurement; TLI, Tucker-Lewis index; YSQ, Yale Single Question

## Figures and Tables

**Fig. 1:**
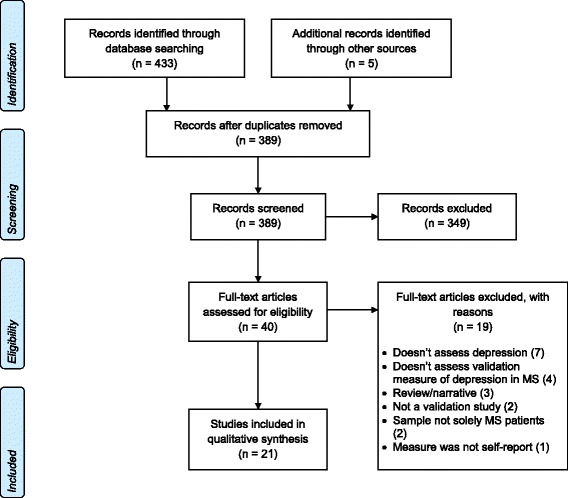
Flow Diagram of Study Selection

## Appendix 2

**Table 6:** Studies Excluded at Full Text Screening, With Reasons

Authors (Date)	Reason for Exclusion
Quaranta et al. (2012) [1]	Measure was not self-report
Knox (2010) [2]	Review/narrative
Manoj & Sivan (2007) [3]	
Nocentini (2006) [4]	
Doward et al. (2009) [5]	Doesn’t assess depression
Fishman et al. (2004) [6]	
Groom et al. (2003) [7]	
McGuigan & Hutchinson (2004) [8]	
Mueller & Girace (1988) [9]	
O’Brien et al. (2007) [10]	
Schwartz et al. (2011) [11]	
Cook et al. (2012) [12]	Doesn't assess validation of depression measure in MS
Gold et al. (2003) [13]	
Horton et al. (2010) [14]	
Provinciali et al. (1999) [15]	
Alajbegovic et al. (2009) [16]	Not a validation study
Good et al. (1992) [17]	
Leon et al. (2001) [18]	Sample not solely PwMS
Lykouras et al. (1998) [19]	

## Appendix 3

**Table 7:** Study Characteristics

Authors (Date), Country	Samples	Baseline Characteristics	MS Diagnosis (years)	Disability Status	Recruitment Method	Measures	Validity/ Reliability Tested
Aikens et al. (1999) [20], USA	MS=105, Depressed=34, Healthy=80, Diabetes=71, Chronic pain=80	MS: *M*_age_=41.9(*SD*= 9.0), 63% female; Depressed: *M*_age_=39.3 (*SD*=14.6), 65% female; Healthy controls: *M* age *M*_age_=34.4 (*SD*=8.3), 50% female; Diabetes: *M*_age_=55.9 (*SD*=16.7), 56% female; Chronic pain: *M*_age_=45.0 (*SD*=13.9), 56% female	*M* = 11 years	Expanded Disability Status Scale = 0.0 - 7.5 (Median = 3.8) Moderate Disability	University Hospital	BDI-II	Construct validity; content validity; internal reliability (cronbach's alpha)
Amtmann et al. (2014) [21], USA	MS = 455	*M*_age_=52.9 (*SD*=10.8); 83% female (*N*=377) and 17% male (*N*=78);	*M* = 14.5 (*SD* = 10)	Moderate level	University Hospital/ National Multiple Sclerosis Society charter	CESD–10, PHQ-9 PROMIS-D-8	Evaluation of dimensionality; inter-item correlation; discriminant/convergent validity
Avasarala et al. (2003) [22], USA	MS=120	Ages: 20–29 *N*=6, 30–39 *N*=25, 40–49 *N*=53, 50–59 *N*=32, 60–69 *N*=4; 71% female	NA	NA	University Hospital	YSQ	Criterion validity
Beeney and Arnett (2008) [23], USA	3 year follow-up MS=52; cross-sectional analysis, MS=96.	*N*=52: *M*_age_=46.57 (*SD*=7.61); *N*=96: *M*_age_=47.41 (*SD*=8.98).	*M* (T1) =14.04 (*SD* = 9.37); *M* (T2) = 16.87 (*SD* = 9.24)	EDSS (T1) =4.55 (*SD* = 1.44) EDSS (T2) = 4.71 (*SD* = 1.61)	University Hospital/ National Multiple Sclerosis Society charter	CMDI	Construct validity
Benedict et al. (2003) [24], USA	MS=54	*M*_age_=42.8 (*SD*=9.7), 79% female	NA	EDSS median = 2.5 (*R* = 0.0 - 7.0)	University Hospital	BDI-FS	Criterion and construct validity
Chang et al. (2003) [25], USA	MS=433; plus 'standardisation sample' *n*=420	MS: *M*_age_=45.0 (*SD*=10.0), 69.4% female; Standardisation sample described in Nyenhuis et al. (1998) [26]: *M*_age_=43.1	NA	NA	University Hospital	CMDI	Content validity, internal reliability, construct validity
Honarmand and Feinstein (2009) [27], Canada	Study 1: MS=140; Study 2: MS=40, MD=21, Matched controls (no MD) = 19	*M*_*age*_=44.6 (*SD*=10.3), 75% female	*M* = 8.8 (*SD* = 6.8)	EDSS = 4.0 (*SD* = 2.34)	Hospital	HADS	Criterion validity
Mohr et al. (1997) [28], USA	MS =184, DEP =72, controls (college students) = 555	MS: *M*_*age*_=44.0, 68% female; DEP: *M*_*age*_=47.5, 51% female; Controls: *M*_*age*_=20.2, 55%	*NA*	NA	University Hospital	BDI	Face validity
Mohr et al. (2007) [29], USA	MS = 260	*M*_*age*_=51 (*SD*=10.5), 73% female	*M* = 19 (*SD* = 10.5)	NA	University Hospital	Two-item measure	Criterion validity, construct validity
Moran and Mohr (2005) [30], USA	MS (with depression)=42	M_age_=43.0 (*SD*=10.3), 69% female	*M* = 6.6 (*SD* = 6.1)	NA	University Hospital/ referrals/ National Multiple Sclerosis Society Charter	BDI	Construct validity
Nicholl et al. (2001) [31], UK	MS=88	*M*_*age*_=48.97 (*SD*=8.9), 75% female	*M* = 11.8 (*SD* = 7.5)	NA	Hospital/Rehabilitation ward	HADS	Criterion validity, construct validity
Nyenhuis et al. (1995) [32], USA	MS=84, DEP=101, controls (MS matched)=87	MS: *M*_*age*_=49.3 (*SD*=11.1), 75% female; DEP: *M*_*age*_=50.5 (*SD*=10.7), 65% female; Controls: *M*_*age*_=49.6 (*SD*=11.6), 75% female	NA	EDSS = 4.74 (*SD* = 3.6)	Community based	BDI, MDI	Construct validity
Pandya et al. (2005) [33], Canada	MS=47	*M*_*age*_=39.3 (range 18–56), 72.3% female	NA	EDSS = 3.0	University hospital/referrals to Psychiatric care	CES-D	Criterion validity, construct validity
Patten et al. (2005) [34], Canada	MS=567	*M*_*age*_=48 (Range 19–76), 75.7% female	NA	NA	University Hospital	CES-D	Construct validity
Patten et al. (2010) [35], Canada	year 0 *N*=1670 year 1; *N*=1336 year 2; *N*=648, year 3 *N*=186	15.9% aged 18–34, 29.5% aged 35–44, 33.6% aged 45–54, 21.0% aged 55+; 77.1% female	NA	EDSS (mode) = 4 (*R* = 4–8)	University Hospital	CES-D	Test-retest reliability
Sjonnesen et al. (2012) [36], Canada	MS=173, Controls (general population)=3304	MS: *M*_*age*_=52.9 (95%CI 51.2–54.6), 74.6% female; Controls: *M*_*age*_=44.4 (95%CI 44.0–44.8), 67.7% female	*M* = 14.4 (95%CI = 13–15.8)	27.1% (46/170) unable to work	Patient Registry/ Hospital	PHQ-9	Content validity, construct validity, internal reliability
Solari et al. (2003) [37], Italy	MS=213, Healthy controls (matched to MS)=213, DEP=32	MS: *M*_*age*_=38 (*SD*=9.2), 66% female; DEP: *M*_*age*_=51.8 (*SD*=14.15), 78% female; Healthy controls: *M*_*age*_=38.3 (*SD*=9.4), 55.9% female.	*M* = 9.1 (*SD* = 6.9)	EDSS = 2.9 (*SD* = 1.6)	University Hospital	CMDI	Internal reliability, test-retest reliability, content validity, construct validity, criterion validity
Strober and Arnett (2010) [38], USA	MS-DEP=17, MS-NON-DEP=67, healthy controls=22	MS-DEP: *M*_*age*_=45.24 (*SD*=8.39), 82% female; MS-NON: *M*_*age*_=47.93 (*SD*=9.30), 84% female; Controls: *M*_*age*_=46.18 (*SD*=13.36), 82% female.	*M* depressed - 10.59 (*SD* = 6.42) *M* MS-NON = 11.15 (*SD* = 8.66)	EDSS depressed = 5.18 (*SD* = 1.5) EDSS MS- NON= 4.32 (*SD* = 1,54	National Multiple Sclerosis Society charter	mBDI	Construct validity
Sullivan et al. (1995) [39], Canada	MS=46	*M*_*age*_=34.4, 78% female	NA	NA	Hospital/ referrals	BDI	Criterion validity, construct validity
Vahter et al. (2007) [40], Estonia (from Manoj and Sivan, 2007 [31])	MS=134	*M*_*age*_=43.8 (*SD*=12.4), 73.9% female	*M* = 9.9 (*SD* = 8.5)	EDSS = 5.8 (*SD* = 2.5)	Hospital	One-item measure	Criterion validity
Verdier-Taillefer et al. (2001) [41], France	MS=857, GP patients=1598, healthy workers=403	MS: *M*_*age*_=47.0 (*SD*=7.2), 63.2% female; GP patients: *M*_*age*_=44.6 (*SD*=8.8), 59.1% female; Healthy workers: *M*_age_=42.9 (*SD*=6.3), 55.3% female	NA	NA		CES-D	Content validity, internal reliability, construct validity

Note: *MS* Multiple Sclerosis, *MD* Major Depression, *DEP* Depressed, *NA* Not Available
